# Supplementary material for: MetaRibo-Seq measures translation in microbiomes
Source: Nat Commun. 2020 Jun 29;11:3268. doi: 10.1038/s41467-020-17081-z (PMC7324362; doi:10.1038/s41467-020-17081-z)
Supplement: Supplementary file 10 — Supplementary Data 7 [file 41467_2020_17081_MOESM10_ESM.zip › File2/Confidence_High_Taxonomy/157831_out.krona.html]

Javascript must be enabled to view this page.

members
magnitude
magnitudeUnassigned
count
unassigned
taxon
rank

157831\_out

6

superkingdom
6
2

phylum
6
1239

class
186801
5

5
186802
order

family
1
31979

1
1485
genus

species
59620
1

SRS014235\_contig\_number\_36043

species

SRS015578\_contig\_number\_23599SRS019397\_contig\_number\_2227SRS043411\_contig\_number\_10061SRS065504\_contig\_number\_20479
4
1898207

1
2292896

SRS142503\_contig\_number\_3088
species
